# Preferential Circularly Polarized Luminescence from a Nano-Segregated Liquid Crystalline Phase Using a Polymerized Twisted Nematic Platform

**DOI:** 10.3390/polym12112529

**Published:** 2020-10-29

**Authors:** Jae-Jin Lee, Suk-Won Choi

**Affiliations:** Department of Advanced Materials Engineering for Information and Electronics, Kyung Hee University, Yongin-shi, Gyeonggi-do 17104, Korea; jjking1443@naver.com

**Keywords:** liquid crystals, reactive mesogens, bent-core molecules, polymerized networks, circularly polarized luminescence, helical nanofilaments

## Abstract

In this study, a polymerized twisted nematic (TN) network was used as an extrinsic chiral platform to overcome the heterogeneity during spontaneous symmetry breaking in a mixed system comprising an achiral bent-core molecule and rod-like mesogen. The TN platform was prepared by photopolymerizing a reactive mesogen dispersed in a low molecular weight liquid crystal with TN orientation. The use of TN orientation to correct the degeneracy in bent-core molecular systems has been previously reported; however, to the best of our knowledge, this is the first study that uses an extrinsic chiral platform of a polymerized TN network. The heterogeneity in the nano-segregated phase of the achiral mixture was suppressed using the extrinsic TN platform with a twisted angle θ of ≥ |±30°|. When an achiral mixture doped with a luminescent guest molecule was refilled into the extrinsic chiral platform, preferential deracemization with one-handedness occurred, corresponding to the handedness of the TN platform. Therefore, circularly polarized luminescence with a preferential handedness can be achieved using this extrinsic chiral platform.

## 1. Introduction

Liquid crystal (LC) phases formed from achiral bent-core (BC) molecules differ from the conventional phases of rod-like mesogens [1,2]. In mixed systems comprising BC molecules and rod-like mesogens, the nano-sized phase segregation occurs between the helical nanofilaments (HNFs), originating from the BC molecules and calamitic LC phases originating from the rod-like mesogens [3,4,5]. This nano-segregated phase shows significant optical rotatory power or circular dichroism [6,7]. Hence, such mixed systems have attracted attention as promising chiroptical materials [8,9,10].

Recently, a study demonstrating an approach to generate circularly polarized luminescence (CPL) using the aforementioned mixed system was reported [11]; CPL was successfully achieved from the nano-segregated phase doped with a luminescent dye. A rod-like mesogen doped with the dye and segregated from the HNFs forms a chiral superstructure; thus, CPL originating from the dye molecules doped in the rod-like mesogen can be observed [11]. This approach enables a simple molecular design to fabricate materials that show CPL from mixed systems consisting of only chemically achiral molecules, because chirality at the molecular level is not necessary. However, non-preferential CPL with left- or right-handedness is observed owing to the degeneracy of the helix handedness of nanofilaments derived from the achiral BC molecules. This is because the achiral BC molecules self-organize into two chiral domains comprising HNFs with opposite helical handedness owing to the lack of chirality at the molecular level [3,12].

Herein, we propose a method of using an extrinsic chiral platform to overcome the aforementioned disadvantage. We fabricated an extrinsic twisted nematic (TN) platform using a photopolymerizable reactive mesogen. The polymerized TN network was used as the extrinsic chiral platform to achieve deracemization during spontaneous symmetry breaking of an achiral system. When this extrinsic chiral platform was refilled with a nano-segregated phase, preferential deracemization with one-handedness, corresponding to the handedness of the TN platform, was achieved. This resulted in CPL with preferential handedness. The use of TN orientation to eliminate the degeneracy of BC molecular systems has been reported previously [13,14]; however, to the best of our knowledge, this is the first study that uses an extrinsic chiral platform derived from a polymerized TN network.

## 2. Experimental

### 2.1. Fabrication of the Extrinsic Chiral Platform

**Figure 1:**
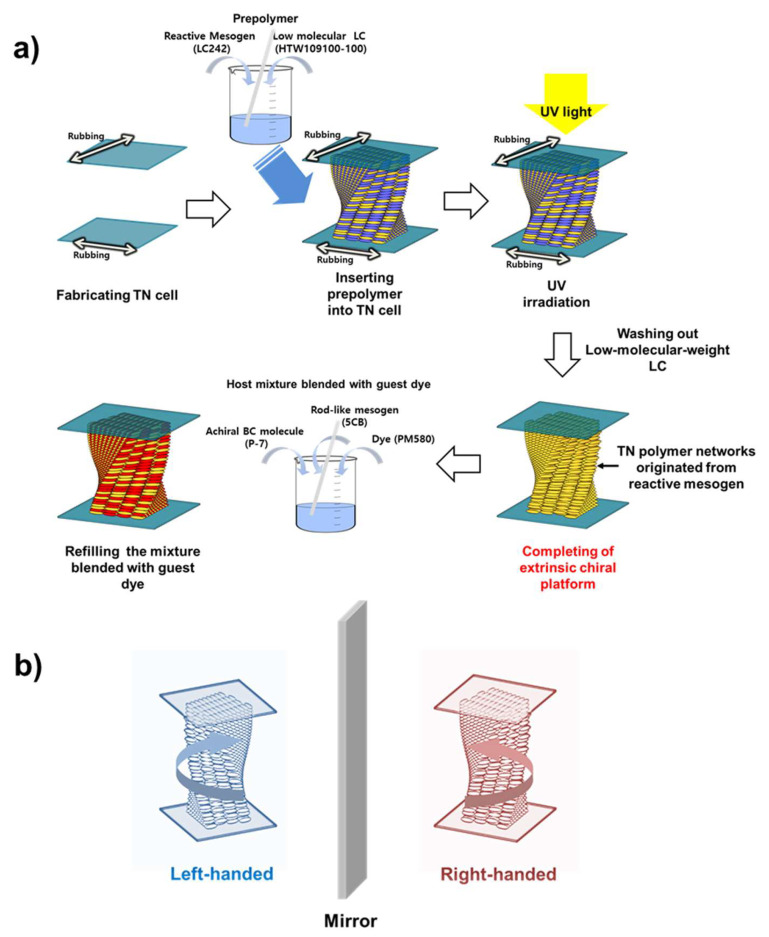
(**a**) Fabrication of the extrinsic chiral platform. (**b**) The pair of twisted nematic (TN) orientations.

**Figure 2:**
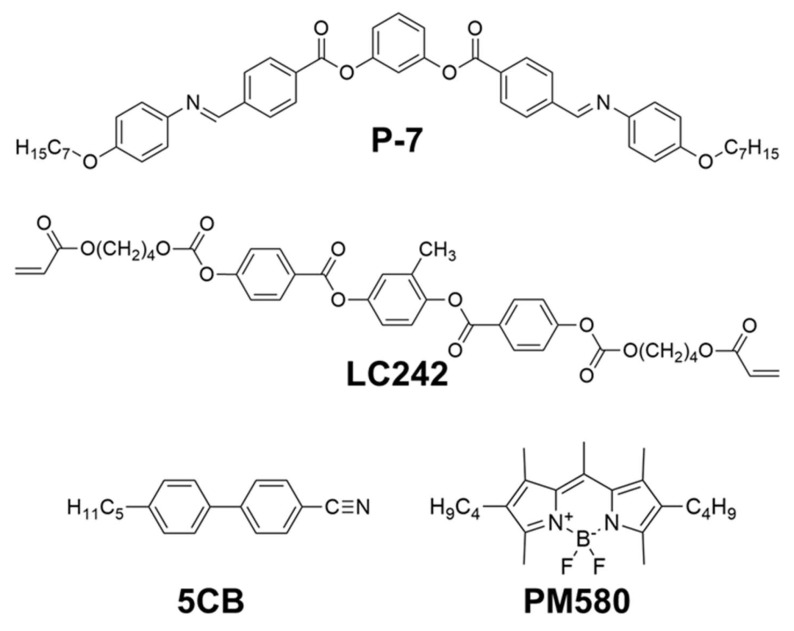
Chemical structures of P-7, LC242, 5CB, and PM580 used in this study.

The fabrication of the extrinsic chiral platform is depicted in Figure 1a. The platform was prepared by photopolymerizing a reactive mesogen dispersed in a low-molecular-weight LC (see Figure 2 for its molecular structure) with TN orientation. Initially, we fabricated TN cells using a conventional LC alignment technique. We prepared thin cells (2 μm in height) and maintained the thickness using ball spacers to prevent the additional optical effect due to the increase in the cell thickness. Optical effects such as the wave-guiding effect of TN cells were not considered because the TN configuration is only used as a chiral template in this case. Only cells with a twisted angle of less than 90° were prepared, because two inversely twisted domains could be generated in the TN cells at a twisted angle θ = 90°. A prepolymer LC mixture consisting of a reactive mesogen (30 wt %, LC242, BASF, Ludwigshafen, Germany), commercial low-molecular-weight LC (70 wt %, HTW109100-100, HCCH, Jiangsu, China), and a small amount of photoinitiator (Sigma-Aldrich, Yongin, Korea) was then inserted into the TN cells. Subsequently, the cells were irradiated with UV light (254 nm, 30 mW cm^−2^) at room temperature (RT), resulting in TN polymer networks originating from the reactive mesogen dispersed in the low-molecular-weight LC. The cells were then immersed in an organic solvent (1,2-dichlorobenzene) at RT to wash out the low-molecular-weight LC and any residual unpolymerized reactive mesogen, and only the polymer TN networks remained. As shown in Figure 1b, the TN orientation pair possesses a chiral structure, because it is distinguishable from its mirror image. Therefore, the cell with the remaining polymer TN network can be regarded as the extrinsic chiral platform. Finally, the cell was refilled with a host mixture composed of an achiral BC molecule (P-7, synthesized in our laboratory) and rod-like mesogen (5CB, Sigma-Aldrich, Seoul, Korea), and then blended with a guest luminescent dye (PM580, Exciton, Lockbourne, Ohio, USA). The host mixture blended with the guest dye was then inserted into the cell in an isotropic state, and the cell was cooled slowly to RT. The chemical structures of P-7, 5CB, and PM580 are depicted in Figure 2.

### 2.2. CPL Measurement

For CPL measurement, a 525 nm light source was employed as the pumping source, and the emitted light (560–580 nm) was modulated with a photoelastic modulator (PEM). To analyze the phase of the CPL signal, the CPL was converted to linearly polarized light and detected by a photomultiplier tube (PMT) after being passed through a linear polarizer. The AC component of the PMT output was analyzed with a lock-in amplifier locked with the reference frequency signal from the PEM. The values of (*I*_left_ − *I*_right_) and (*I*_left_ + *I*_right_) were deduced from the AC and DC components of the modulated signal as a function of wavelength. The luminescence dissymmetry factor (g_lum_) was evaluated using the following equation:

g_lum_ = 2(*I*_left_ − *I*_right_)/(*I*_left_ + *I*_right_)) (1)

where *I*_left_ and *I*_right_ are the magnitudes of the left and right circularly polarized light components, respectively. The details of the CPL measurement are described elsewhere [10,11,15].

## 3. Results and Discussion

**Figure 3:**
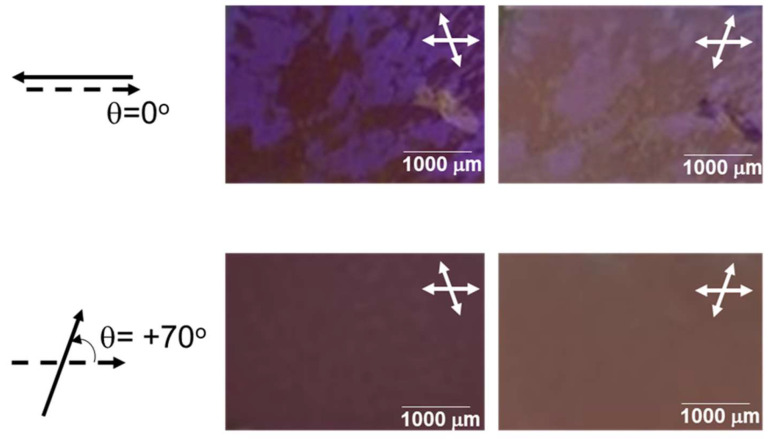
Typical polarized optical microscopy (POM) images displaying the textures of the refilled binary mixture in the cells with extrinsic TN platforms, at a twisted angle θ = 0° (antiparallel) and θ = +70° (corresponding to a clockwise, left-handed twist) under slightly decrossed polarizers.

Figure 3 shows the typical polarized optical microscopy (POM) images that display the textures of the refilled binary mixture (P-7:5CB = 40:60 wt %) in the cells with extrinsic TN platforms at a twisted angle θ = 0° (antiparallel) and θ = +70° (corresponding to a clockwise, left-handed twist) under slightly decrossed polarizers. The phase sequences of pure P-7 and 5CB upon cooling are denoted as Iso-170 °C–B2-155 °C–B7-144°C–HNF and Iso-35 °C–Nematic (N)-23 °C–Cryst, respectively. Upon mixing the BC molecule (P-7) with the rod-like mesogen (5CB), nanoscale phase segregation occurred; therefore, the refilled binary mixture showed the following phase sequences upon cooling: Iso- 125 °C -<HNF/Iso>-33 °C -<HNF/N>. Herein, <HNF/Iso> indicates a nano-segregated phase, in which the BC molecules are in the HNF phase, and rod-like molecules are in the Iso phase of the mixture. Similarly, <HNF/N> indicates a nano-segregated phase in which the BC molecules are in the HNF phase and rod-like molecules are in the N phase [16]. The POM images were obtained at RT; thus, the nano-segregated <HNF/N> phase was observed. For the cell with a twisted angle θ = 0°, the two randomly distributed chiral domains was apparent, and the brightness of the two domains interchanged when the polarizer was decrossed in the opposite direction. This heterogeneity occurred due to the two unbiased helix directions of the HNFs originating from the achiral BC molecules. In contrast, for the cell with a twisted angle θ = +70°, one chiral domain was observed across the entire area under survey. This is because the extrinsic TN platform biased the formation of a particular chiral arrangement of the HNFs. In this study, the heterogeneity in the <HNF/N> phase was suppressed by the use of the extrinsic TN platform, with a twisted angle θ≥|±30°|. Note that if perfect deracemization with one-handedness is achieved, further enhancement of g_lum_ can be expected.

**Figure 4:**
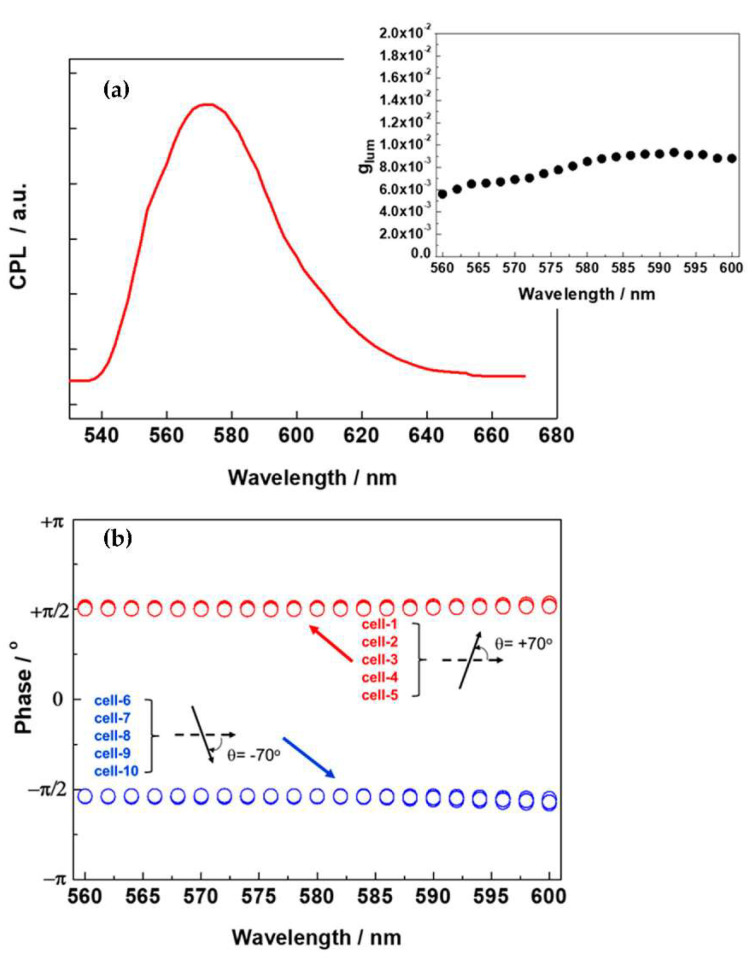
(**a**) A typical CPL spectrum obtained from cells with the extrinsic TN platform at a twisted angle of θ = +70°. The calculated g_lum_ values are depicted in the inset plot; (**b**) phase (−π/2 or +π/2) of the modulated CPL signal from the cells with extrinsic TN platforms with a twisted angle θ= −70° and θ = +70°.

Figure 4a presents a typical CPL spectrum obtained from the cells with extrinsic TN platform at a twisted angle θ = +70°. The calculated g_lum_ values are also depicted in the inset of Figure 4a. Figure 4b shows the phase of the modulated CPL signal from the cells with extrinsic TN platforms, at the twisted angle θ = −70° (corresponding to a counterclockwise, right-handed twist) and θ = +70°. We prepared several cells with twisted angles θ = –70° and +70°, which were refilled with a 98 wt % host mixture (P-7:5CB = 40:60 wt %) and blended with 2 wt % of the guest dye. The measurements were performed at RT, where all the cells exhibited the nano-segregated <HNF/N> phase. As shown in Figure 4b, the phase of the CPL signal emitted from all the cells with a twisted angle θ = −70° (+70°) exhibited − π/2 (+π/2) without any exceptions. Therefore, the phase difference between the cells with the twisted angles θ= −70° and +70° was equal to π, indicating that the corresponding CPL signals had opposite signs, i.e., the CPL from each cell was either right or left circularly polarized. Therefore, we confirmed that preferential CPL with right- or left-handedness from the nano-segregated <HNF/N> phase could be obtained using the extrinsic chiral (TN) platforms with a range of twisted angles (|30°|≤θ≤|80°|). The g_lum_ values obtained from cells with different twisted angles (|30°|≤θ≤|80°|) ranged from 6 × 10^−3^ to 8 × 10^−3^, irrespective of the twisted angle. These results indicate that the CPL is mainly attributable to the chiral superstructure originating from the HNFs (BC molecules), and the extrinsic chiral (TN) platform only influenced the formation of preferential handedness of the HNFs.

The measured g_lum_ values are small to be used in practical applications. Recently, a relatively large g_lum_ value was obtained in a TN configuration using a mesogenic conjugate polymer [17]. The small g_lum_ values of our system are because only achiral low-molecular-weight molecules were used. Further enhancement of g_lum_ is desirable for the practical application of our system [18].

## 4. Conclusions

In this study, a polymerized TN network was used as an extrinsic chiral platform to achieve molecular deracemization during spontaneous symmetry breaking in a mixed system comprising an achiral BC molecule and rod-like mesogen. The heterogeneity of the nano-segregated phase of the achiral mixture was suppressed using extrinsic TN platforms with a twisted angle θ of ≥ |±30°|. When the achiral mixture doped with a luminescent guest molecule was refilled into this extrinsic chiral platform, preferential deracemization of the achiral mixture occurred with one-handedness, corresponding to the handedness of the TN platform, resulting in CPL with a preferential handedness. Due to the preferential helicity of the HNF, the structure of the embedded LC phase doped with the luminescent guest-formed self-assembled chiral aggregates associated with the helix of the HNF phase. Thus, our extrinsic chiral platform could potentially be employed for chiral separations and asymmetric synthesis. Our findings could lead to a useful method for achieving preferential deracemization with one-handedness without the need for any chiral species.

## References

[1] Takezoe H., Takanishi Y. (2006). Bent-Core Liquid Crystals: Their Mysterious and Attractive World. Jpn. J. Appl. Phys..

[2] Reddy R.A., Tschierske C. (2006). Bent-Core Liquid Crystals: Polar Order, Superstructural Chirality and Spontaneous Desymmetrisation in Soft Matter Systems. J. Mater. Chem..

[3] Le K.V., Takezoe H., Araoka F. (2017). Chiral Superstructure Mesophases of Achiral Bent-Shaped Molecules—Hierarchical Chirality Amplification and Physical Properties. Adv. Mater..

[4] Takanishi Y., Shin G.J., Jung J.C., Choi S.W., Ishikawa K., Watanabe J., Takezoe H., Toledano P. (2005). Observation of Very Large Chiral Domains in a Liquid Crystal Phase Formed by Mixtures of Achiral Bent-Core and Rod Molecules. J. Mater. Chem..

[5] Chen D., Tuchband M.R., Horanyi B., Korblova E., Walba D.M., Glaser M.A., Maclennan J.E., Clark N.A. (2015). Diastereomeric Liquid Crystal Domains at the Mesoscale. Nat. Commun..

[6] Otani T., Araoka F., Ishikawa K., Takezoe H. (2009). Enhanced Optical Activity by Achiral Rod-Like Molecules Nanosegregated in the B_4_ Structure of Achiral Bent-Core Molecules. J. Am. Chem. Soc..

[7] Jeon S.-W., Kim D.-Y., Araoka F., Jeong K.-W., Choi S.-W. (2017). Nanosegregated Chiral Materials with Self-Assembled Hierarchical Mesophases: Effect of Thermotropic and Photoinduced Polymorphism in Rodlike Molecules. Chem. Eur. J..

[8] Kim K., Kim H., Jo S.-Y., Araoka F., Yoon D.K., Choi S.-W. (2015). Photomodulated Supramolecular Chirality in Achiral Photoresponsive Rodlike Compounds Nanosegregated from the Helical Nanofilaments of Achiral Bent-Core Molecules. ACS Appl. Mater. Interfaces.

[9] Jeon S.-W., Choi H.-J., Bae J.-H., Kim B.-C., Choi S.-W. (2018). Photomodulating Chiroptic Behaviors in Nanosegregated Mesophase from a Mixture System Consisting of Nonchiral Bent-Core and Photo-Responsive Rod-Like Mesogens. J. Inf. Disp..

[10] Lee J.-J., Kim B.-C., Choi H.-J., Bae S., Araoka F., Choi S.-W. (2020). Inverse Helical Nanofilament Networks Serving as a Chiral Nanotemplate. ACS Nano.

[11] Kim B.-C., Choi H.-J., Lee J.-J., Araoka F., Choi S.-W. (2019). Circularly Polarized Luminescence Induced by Chiral Super Nanospaces. Adv. Funct. Mater..

[12] Hough L.E., Jung H.T., Krüerke D., Heberling M.S., Nakata M., Jones C.D., Chen D., Link D.R., Zasadzinski J., Heppke G. (2009). Helical Nanofilament Phases. Science.

[13] Choi S.-W., Kang S., Takanishi Y., Ishikawa K., Watanabe J., Takezoe H. (2006). Intrinsic Chirality in a Bent-Core Mesogen Induced by Extrinsic Chiral Structures. Angew. Chem. Int. Ed..

[14] Ueda T., Masuko S., Araoka F., Ishikawa K., Takezoe H. (2013). A General Method for the Enantioselective Formation of Helical Nanofilaments. Angew. Chem. Int. Ed..

[15] Riehl J.P., Richardson F.S. (1986). Circularly Polarized Luminescence Spectroscopy. Chem. Rev..

[16] Araoka F., Sugiyama G., Ishikawa K., Takezoe H. (2013). Highly Ordered Helical Nanofilament Assembly Aligned by a Nematic Director Field. Adv. Funct. Mater..

[17] Baek K., Lee D.M., Lee Y.J., Choi H., Seo J., Kang I., Yu C.J., Kim J.H. (2019). Simultaneous emission of orthogonal handedness in circular polarization from a single luminophore. Light Sci. Appl..

[18] Lee J.-J., Choi S.-W. (2020). Enhancement of Luminescence Dissymmetry Factor in Nano-Segregated Phase Generated by Phase Separation between Helical Nanofilaments and Liquid-Crystalline Smectic A Phase. Crystals.

